# U-shaped association between central body fat and the urinary albumin-to-creatinine ratio and microalbuminuria

**DOI:** 10.1186/1471-2369-14-87

**Published:** 2013-04-17

**Authors:** Kathleen Dittmann, Anke Hannemann, Henri Wallaschofski, Rainer Rettig, Sylvia Stracke, Henry Völzke, Matthias Nauck, Nele Friedrich

**Affiliations:** 1 Institute of Clinical Chemistry and Laboratory Medicine, Ernst-Moritz-Arndt University of Greifswald, Ferdinand-Sauerbruch-Strasse NK, Greifswald, D-17475, Germany; 2 Institute of Physiology, Ernst-Moritz-Arndt University of Greifswald, Greifswald, Germany; 3 Department of Internal Medicine A, Ernst-Moritz-Arndt University of Greifswald, Greifswald, Germany; 4 Institute for Community Medicine, Ernst-Moritz-Arndt University of Greifswald, Greifswald, Germany

**Keywords:** Anthropometry, Chronic kidney disease, Estimated glomerular filtration rate, Microalbuminuria, Study of Health in Pomerania, Urinary albumin-to-creatinine ratio

## Abstract

**Background:**

The prevalence of obese and overweight patients has increased dramatically worldwide. Both are common risk factors for chronic kidney disease (CKD) as indicated by a diminished estimated glomerular filtration rate (eGFR) or microalbuminuria. This study aimed to investigate whether anthropometric parameters [waist circumference (WC), waist-to-height ratio (WHtR) and body mass index (BMI)] are associated with renal function in a population-based study of Caucasian subjects.

**Methods:**

Data from 3749 subjects (1825 women) aged 20 to 81 years from the Study of Health in Pomerania (SHIP) were analysed. Renal indices, including the urinary albumin-to-creatinine ratio (uACR), microalbuminuria, eGFR and CKD, were studied. Parameters of anthropometry (WC, WHtR and BMI) were categorised into sex-specific quintiles.

**Results:**

Analysis of variance (ANOVA) models, adjusting for age, sex, type 2 diabetes mellitus and hypertension, revealed that a high and low WC or WHtR and low BMI were independently related to a higher uACR. Logistic regression models confirmed these results with respect to uACR and showed that subjects with a high or low WC or a high WHtR had increased odds of microalbuminuria. The ANOVA models revealed no relations of the investigated anthropometric parameters with eGFR. However, subjects with high values for these parameters had increased odds of CKD.

**Conclusions:**

Our results demonstrate U-shaped associations between markers of central fat distribution and uACR or microalbuminuria in the general population, suggesting that both obese and very thin subjects have a higher risk of renal impairment.

## Background

Chronic kidney disease (CKD) is a global public health problem with an increasing incidence and prevalence, poor outcomes and high cost [1]. The prevalence of recognized CKD among subjects in the U.S. national social insurance program Medicare increased three-fold between 2000 and 2009, from 2.7 to 8.5% [2]. In most patients, CKD is silent and commonly not detected until an advanced stage [3]. Being overweight [body mass index (BMI) 25–30 kg/m^2^] and obesity (BMI >30 kg/m^2^) represent major risk factors for renal dysfunction [4,5]. Several studies [6-9] have shown that obesity is a risk factor for a diminished glomerular filtration rate (GFR) and CKD. Data from the Framingham Heart Study [7] revealed that BMI is a strong predictor for developing CKD. A retrospective case–control study [8] confirmed these findings and indicated that young overweight subjects had a three-fold higher risk of stage 5 CKD than lean control subjects.

Moreover, waist circumference (WC) and waist-to-hip ratio (WHR) as indices of visceral obesity have been reported to be even more sensitive predictors of late-stage renal disease than BMI [6]. Data from the Prevention of Renal and Vascular End-Stage Disease (PREVEND) study [9] revealed that obese subjects with a central fat distribution had a higher risk of microalbuminuria than lean subjects with a peripheral fat distribution. Furthermore, that study [9] showed that not only overweight and obese subjects but also lean subjects with central fat distribution were at a higher risk of a diminished GFR.

This study aimed to identify possible associations between WC, WHtR or BMI and renal dysfunction based on the urinary albumin-to-creatinine ratio (uACR), microalbuminuria, estimated GFR (eGFR) and CKD in an adult Caucasian population.

## Methods

### Study population

The Study of Health in Pomerania (SHIP) is a population-based cohort study in West Pomerania, a region in northeastern Germany. Details on the SHIP design, recruitment and procedures have been published elsewhere [10]. Baseline data collection started in October 1997 and was finished in March 2001. The initial sample for the baseline examination consisted of 4308 participants (net response: 69%). All of the participants gave written informed consent. The study conformed to the principles of the Declaration of Helsinki as reflected by an a priori approval by the Ethics Committee of the University of Greifswald. Data usage for the project (number: SHIP/2011/50/D) was approved by the Research Network of Community Medicine and the manuscript was approved by the Publication Committee.

Of the 4308 participants, the 527 subjects with missing values for serum creatinine, urinary creatinine or serum albumin were excluded. Furthermore, 12 pregnant women and 20 subjects with missing values for anthropometric parameters or certain confounding factors were excluded. Altogether, the final study population included 3749 subjects (1924 men; 1825 women) aged 20 to 81 years.

### Data collection

Information on age, sex, sociodemographic characteristics and medical history was collected using computer-assisted personal interviews. The definition of type 2 diabetes mellitus was based on a self-reported physician diagnosis or use of an antidiabetic medication [anatomic, therapeutic and chemical (ATC) code: A10] during the last 7 days. After a five-minute resting period, systolic and diastolic blood pressures were measured three times on the right arm of seated subjects, using a digital blood pressure monitor (HEM-705CP, Omron Corporation, Tokyo, Japan). Each reading was followed by another rest period of three minutes. The mean of the second and third measurements was used for the statistical analyses. Hypertension was defined as systolic blood pressure ≥140 mmHg, diastolic blood pressure ≥90 mmHg or self-reported use of antihypertensive medication.

Height was measured to the nearest 1 cm using a digital ultrasound instrument, and weight was measured to the nearest 0.1 kg in light clothing and without shoes using standard digital scales (Soehnle-Waagen GmbH, Nassau, Germany). WC was measured to the nearest 0.1 cm using an inelastic tape midway between the lower rib margin and the iliac crest in the horizontal plane, with the subject standing comfortably with weight distributed evenly on both feet. The WHtR was calculated as the waist circumference in centimetres divided by the height in centimetres. The BMI was calculated as the weight in kilograms divided by the square of height in metres.

Non-fasting blood and urine samples were collected between 7.00 a.m. and 6.00 p.m. Blood samples were taken from the cubital vein of subjects in the supine position and prepared for immediate analysis or for storage at −80°C for further analysis. Serum creatinine was measured using the Jaffé method (Hitachi 717; Roche Diagnostics, Mannheim, Germany). Urinary creatinine and albumin concentrations were determined using a Behring Nephelometer (Siemens BN albumin; Siemens Healthcare, Marburg, Germany) and a Hitachi 717 device (Roche Diagnostics), respectively. The uACR was calculated using the following equation: uACR (mg/mmol) = urinary albumin concentration (mg/L)/urinary creatinine concentration (mmol/L). Microalbuminuria was defined as an uACR ≥2.5 mg/mmol in men or ≥3.5 mg/mmol in women [11].

The eGFR was calculated using the four-variable Modification of Diet in Renal Disease (MDRD) study equation: eGFR = 186.3 * serum creatinine^-1.154^ * age^-0.203^ * (0.742 if female) [1,12]. CKD was defined as an eGFR <60 mL/min/1.73 m^2^, consistent with the definition of CKD ≥ stage 3 proposed by the National Kidney Foundation Kidney Disease Outcomes Quality Initiative (KDOQI) [1].

### Statistical analyses

Continuous data are expressed as the median (25th; 75th quartile) and nominal data are expressed as a percentage. For bivariate comparisons between women and men for the baseline characteristics of the study population, the Kruskal-Wallis test (continuous data) or the χ^2^-test (nominal data) was used. As a first step, linear regression with restricted cubic splines [13] was used to detect the possible dependency of eGFR or uACR on WC, WHtR or BMI. Three knots were pre-specified, located at the 5^th^, 50^th^ and 95^th^ percentile, as recommended by Stone and Koo [13], and the uACR values were log-transformed. Based on these results, the WC, WHtR and BMI values were categorised into five groups according to sex-specific quintiles of distribution (Additional file 1: Table S1).

Next, analysis of variance (ANOVA) models were used to test for differences in the uACR or eGFR among sex-specific quintiles of WC, WHtR or BMI. Logistic regression models were performed to assess the associations between parameters of anthropometry and microalbuminuria or CKD. All models were adjusted for age, sex, type 2 diabetes mellitus and hypertension. Furthermore, we tested whether age or sex had a potential effect on our ordinal regression models, and no significant interaction (p < 0.10) between age and the tested variable was found. Regarding the interaction between sex and the tested variable, only one out of six models detected a significant interaction (WHtR and microalbuminuria). Therefore, we decided not to perform age- or sex-stratified analyses. Sensitivity analyses were performed after excluding subjects with type 2 diabetes mellitus or subjects who died within the first 5 years of follow up. Adjusted means and odds ratios (ORs) with 95% confidence intervals (CIs) were calculated. A p-value <0.05 was considered to be statistically significant. Statistical analyses were performed using SAS 9.2 (SAS Institute Inc., Cary, NC).

## Results

### General characteristics

In our study population, the men were older and more often had hypertension than the women (Table 1). Regarding anthropometric parameters, the men were taller and had higher values for weight, WC, WHtR and BMI than the women. With respect to kidney function, the men more often had microalbuminuria and a higher eGFR, and less often had CKD, albeit they had higher serum creatinine concentrations than the women.

**Table 1:** Characteristics of the study population

**Characteristics**	**Men (n = 1924)**	**Women (n = 1825)**	**p-value**
Age (years)	52 (37; 65)	49 (35; 62)	<0.01
Type 2 diabetes mellitus (%)	8.8	8.1	0.42
Hypertension (%)	62.9	41.8	<0.01
Weight (kg)	83.9 (75.8; 92.7)	68.8 (60.9; 79.1)	<0.01
Height (cm)	175 (170; 180)	163 (158; 167)	<0.01
Waist circumference (cm)	95.5 (87.5; 103.0)	81.5 (73.0; 92.1)	<0.01
WHtR	0.55 (0.50; 0.60)	0.50 (0.44; 0.57)	<0.01
BMI (kg/m^2^)	27.4 (24.9; 30.0)	26.2 (22.8; 30.2)	<0.01
Serum creatinine (mg/dL)	1.02 (0.94; 1.11)	0.87 (0.80; 0.94)	<0.01
Urinary albumin (mg/L)	8.5 (4.8; 18.3)	7.2 (3.9; 14.5)	<0.01
Urinary creatinine (mmol/L)	10.0 (6.8; 13.8)	6.7 (4.1; 10.3)	<0.01
uACR (mg/mmol)	0.83 (0.51; 1.87)	1.06 (0.66; 2.00)	<0.01
Microalbuminuria (%)	19.3	14.5	<0.01
eGFR (mL/min/1.73 m^2^)	83.24 (73.92; 93.00)	74.81 (66.66; 84.46)	<0.01
CKD (%)	6.5	11.4	<0.01

BMI = body mass index; CKD = chronic kidney disease; eGFR = estimated glomerular filtration rate; uACR = urinary albumin-to-creatinine ratio; WHtR = waist-to-height ratio.

### Urinary albumin-to-creatinine ratio and estimated glomerular filtration rate

**Figure 1:**
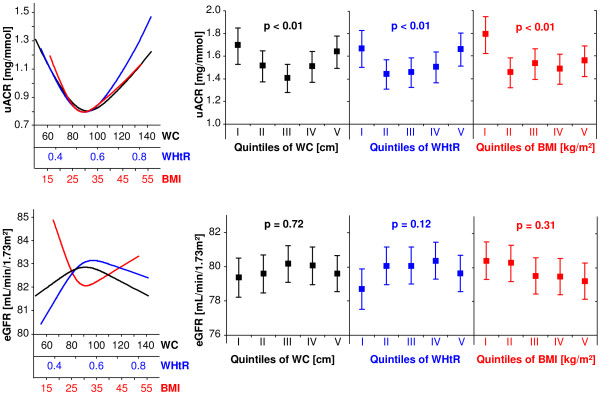
**Association between waist circumference (WC), waist-to-height ratio (WHtR) or body mass index (BMI) and uACR (upper panels) or eGFR (lower panels) for the whole study population.** Left side: Linear regression with restricted cubic splines. Right side: Adjusted mean uACR and adjusted mean eGFR with 95% confidence intervals according to sex-specific quintiles of WC, WHtR or BMI calculated by analyses of variance. All models were adjusted for age, sex, type 2 diabetes mellitus and hypertension.

First, linear regression models with restricted cubic splines revealed U-shaped associations between WC, WHtR or BMI and the uACR or eGFR (Figure 1, *left panels*). Multivariable ANOVA models confirmed these U-shaped relationships between WC or WHtR and uACR (Figure 1, *upper right panel*). Specifically, subjects in the lowest and highest WC and WHtR sex-specific quintiles had up to 0.29 mg/mmol higher uACRs than the subjects in the middle quintile. With respect to BMI, only subjects within the lowest group had higher uACRs than the remaining groups (uACRs in the lowest and highest BMI groups were 1.79 mg/mmol and 1.56 mg/mmol, respectively). No associations were detected between the investigated anthropometric parameters and eGFR (Figure 1, *lower right panel*).

### Microalbuminuria and chronic kidney disease

Logistic regression models were performed to assess the associations between anthropometric parameters and microalbuminuria or CKD. The analyses confirmed the U-shaped association between WC and microalbuminuria (Table 2). Subjects with a high or low WC had up to a 52% increased odds of microalbuminuria compared to the subjects in the middle quintile WC group. Furthermore, a high WHtR was related to increased odds of microalbuminuria, whereas the relationship between a low WHtR and microalbuminuria was not statistically significant. With respect to CKD, subjects in the highest quintiles for WC, WHtR or BMI exhibited up to 83% increased odds of CKD compared to subjects in the middle quintile group. No relationship was found between low anthropometric parameters and CKD.

**Table 2:** Logistic regression models for the associations between sex-specific quintiles of WC, WHtR or BMI and microalbuminuria or CKD in the whole study population (n = 3749)

	**Microalbuminuria**		**CKD**	
	**OR (95% CI)**	***p-value***	**OR (95% CI)**	***p-value***
***Waist circumference (WC) [cm]***				
I	**1.48 (1.06; 2.06)**	**0.02**	1.26 (0.75; 2.11)	0.38
II	1.14 (0.84; 1.55)	0.40	1.12 (0.73; 1.71)	0.60
III	*reference*		*reference*	
IV	1.21 (0.91; 1.60)	0.18	1.19 (0.83; 1.71)	0.35
V	**1.52 (1.16; 2.00)**	**<0.01**	**1.63 (1.15; 2.32)**	**<0.01**
***Waist-to-height ratio (WHtR)***				
I	1.20 (0.85; 1.71)	0.30	1.56 (0.87; 2.78)	0.14
II	0.91 (0.66; 1.24)	0.54	1.39 (0.89; 2.17)	0.14
III	*reference*		*reference*	
IV	1.06 (0.80; 1.39)	0.69	1.18 (0.81; 1.72)	0.39
V	**1.33 (1.03; 1.74)**	**0.03**	**1.83 (1.28; 2.60)**	**<0.01**
***Body mass index (BMI) [kg/m***^***2***^***]***				
I	1.28 (0.94; 1.75)	0.11	0.92 (0.57; 1.48)	0.72
II	0.91 (0.68; 1.23)	0.56	0.86 (0.56; 1.31)	0.48
III	*reference*		*reference*	
IV	1.01 (0.76; 1.33)	0.96	1.00 (0.70; 1.44)	0.99
V	1.16 (0.89; 1.53)	0.27	**1.47 (1.04; 2.10)**	**0.03**

CI = confidence interval; CKD = chronic kidney disease; OR = odds ratio.

Our main results were confirmed in several sensitivity analyses after excluding all subjects with type 2 diabetes mellitus (316 subjects), subjects who died within the first 5 years of follow-up, which indicates critical illness (259 subjects), or subjects with sample collection after noon (1037 subjects, Additional file 1: Table S2). Furthermore, HDL-cholesterol and total cholesterol were tested as further confounders (Additional file 1: Tables S3 and S4). All these analyses confirmed the main results and showed U-shaped associations between the considered anthropometric parameters and uACR as well as a positive relation between anthropometry and CKD.

## Discussion

In this study, we observed associations between three different parameters of anthropometry and uACR or microalbuminuria. Both a low and high WC, WHtR and BMI positively correlated with the uACR. Furthermore, a high or low WC and a high WHtR were associated with increased odds of microalbuminuria. In contrast to the uACR, no association between any of the considered anthropometric parameters and eGFR was detected. However, subjects with high anthropometric values had higher odds of stage 3 CKD than subjects with average values.

In a previous study [14], obesity was related to an increased albumin excretion rate. Interestingly, in obese subjects with the same percentage of total body fat, the risk of abnormal albumin excretion was 18 times higher in those with central obesity but only four times higher in subjects with peripheral obesity compared to controls [14]. The PREVEND study [9] similarly showed that obese subjects with central fat distribution defined by an increased waist-to-hip ratio (WHR ≥0.9 for men and ≥0.8 for women) had an increased risk of microalbuminuria compared to obese subjects with a peripheral fat distribution (WHR <0.9 for men and <0.8 for women). Furthermore, not only obese but also lean or overweight subjects with a central fat distribution were at a higher risk of a diminished GFR than subjects with a peripheral fat distribution [9].

We also observed positive associations between high WC, WHtR or BMI and the risk of CKD. These findings confirm the results of a community-based, cross-sectional study [15] that found a positive relationship between WHtR and the risk of CKD. There was no association between BMI and CKD in this study [15]. These results support the conclusion that WC [6] and WHtR [15] are more sensitive markers of CKD than BMI.

A study in 143 children with CKD [16] revealed that advanced CKD was associated with a low lean leg mass, indicating skeletal muscle wasting. Recent literature [17] suggests that muscle wasting in the context of CKD may be due to inflammation. Another study [18] showed that patients with a relatively modest degree of CKD are characterised by reduced lean body mass and bone mineral content. These findings reveal the complex relationships between anthropometry, metabolism and renal function and indicate that fat tissue measurement may contribute to the detection of renal impairment.

Central obesity is frequently associated with risk factors of CKD, such as hypertension [19,20] and type 2 diabetes mellitus [20,21]. The PREVEND study [9] described higher systolic and diastolic blood pressures in overweight and obese subjects with a central fat distribution than in subjects with a peripheral fat distribution. During the last decades, adipose tissue has emerged as an active endocrine organ that releases several bioactive mediators, modulating haemostasis, blood pressure, lipid and glucose metabolism, inflammation and atherosclerosis [21]. Therefore, our findings of an association between WHtR or WC, as indices of central obesity [22], and the risk of microalbuminuria argue for an important role for visceral vs. peripheral fat in relation to albumin excretion.

Previous studies suggest that abdominal obesity may independently associate with microalbuminuria [9,14,23-27]. Data from the MONICA Augsburg survey 1994/95 [23] showed that microalbuminuric subjects had a higher WC, WHR and prevalence of elevated central adiposity and obesity than subjects without microalbuminuria. Another study [28] using measurements of visceral adipose tissue (VAT) and subcutaneous adipose tissue (SAT) revealed that VAT was associated with microalbuminuria in men but not in women and showed that microalbuminuria may represent a manifestation of visceral adiposity. However, our observation of an association between a low WC and risk of microalbuminuria argues against that assumption. Reason for these discrepancies might be the different definitions of obesity as well as microalbuminuria. Obesity might be based on WC, WHR, WHtR or BMI, which all do not differentiate between VAT and SAT accumulation. Furthermore, altered techniques used for the detection of microalbuminuria resulting in a reported prevalence of microalbuminuria ranging from 10% to 40% [29,30].

The pathophysiology underlying the association between low anthropometry and microalbuminuria is yet unknown. Which is partly part due to the fact that the pathophysiology leads to the development of microalbuminuria are not fully understood. The most likely reason is an alteration of intrarenal hemodynamics and may represent in hypertension as well as type 2 diabetes mellitus as an early unspecific feature of renal impairment. Even if the relation between low anthropometry and microalbuminuria is not fully explained, it is widely accepted that the association between BMI and all-cause mortality was U-shaped with the lowest mortality between 22.5-25 kg/m^2^[31]. Thus a low or high BMI is related to higher mortality risk. Furthermore, there is no doubt that microalbuminuria is a valid biomarker of increased mortality risk [32]. Therefore, microalbuminuria in low values of anthropometry might reflect a damage of glomerulus with complete different pathophysiology compared to vascular risk burden, like hypertension or diabetes, in obese people. There is good evidence of epidemiological studies that lowering fetal growth or low birth weight, result in an increased incidence of hypertension or renal impairment when the offspring reaches adulthood. One possible hypothesis might be, that conditions affecting fetal development leading to low birth weight are associated with renal programming or subclinical renal disease [33]. Microalbuminuria in such people might be one biomarker of these conditions, which is absolutely speculative at the moment.

In general, cross sectional epidemiological studies are not suited to estimate pathophysiology and causality. Therefore, further science, especially animals studies or interventional trials are needed to investigate the phenomenon of U-shaped association between microalbuminuria and anthropometry in more detail.

Two limitations of our study merit special consideration. First, due to the lack of longitudinal data, we cannot detect causality between the anthropometric measures and renal dysfunction. Second, instead of complex and cost-intensive measurements of fat distribution, we used common measurements, such as height, weight and WC to assess the relationship between anthropometric parameters with renal dysfunction. These parameters are easy to measure in clinical practice and public health settings but may not be as accurate as measuring body fat distribution.

## Conclusions

Our data suggest that both a high and low WC or WHtR and low BMI are associated with a higher uACR. Not only subjects with a high WC or WHtR but also subjects with a low WC may have an increased risk of microalbuminuria. However, our data provide no evidence that any of the considered anthropometric parameters are associated with eGFR. Nevertheless, subjects with a high WC, WHtR or BMI exhibit an increased risk of CKD compared to subjects with average anthropometric values.

## Competing interests

The authors declare that they have no competing interests.

## Authors’ contributions

Conception and Design: KD, AH, HW, NF. Data Analysis: KD. Interpretation of data: All. Article drafting: KD, NF, HW. Final approval: All authors read and approved the final manuscript.

## Pre-publication history

The pre-publication history for this paper can be accessed here:

http://www.biomedcentral.com/1471-2369/14/87/prepub

## Supplementary Material

Additional file 1: Table S1 Percentiles of WC, WHtR and BMI in men (n=1924) and women (n=1825). **Table S2.** Logistic regression models for the associations between sex-specific quintiles of WC, WHtR or BMI and microalbuminuria or CKD in the subjects time of examination before noon (n = 2712). **Table S3.** Logistic regression models for the associations between sex-specific quintiles of WC, WHtR or BMI and microalbuminuria (additionally adjusted for HDL-cholesterol and total cholesterol) in the whole study population (n = 3749). **Table S4.** Logistic regression models for the associations between sex-specific quintiles of WC, WHtR or BMI and CKD (additionally adjusted for HDL-cholesterol and total cholesterol) in the whole study population (n = 3749). Click here for file
