# Supplementary material for: U-shaped association between central body fat and the urinary albumin-to-creatinine ratio and microalbuminuria
Source: BMC Nephrol. 2013 Apr 17;14:87. doi: 10.1186/1471-2369-14-87 (PMC3637595; doi:10.1186/1471-2369-14-87)
Supplement: Additional file 1: Table S1 — Percentiles of WC, WHtR and BMI in men (n=1924) and women (n=1825). Table S2. Logistic regression models for the associations between sex-specific quintiles of WC, WHtR or BMI and microalbuminuria or CKD in the subjects time of examination before noon (n = 2712). Table S3. Logistic regression models for the associations between sex-specific quintiles of WC, WHtR or BMI and microalbuminuria (additionally adjusted for HDL-cholesterol and total cholesterol) in the whole study population (n = 3749). Table S4. Logistic regression models for the associations between sex-specific quintiles of WC, WHtR or BMI and CKD (additionally adjusted for HDL-cholesterol and total cholesterol) in the whole study population (n = 3749). [file 1471-2369-14-87-S1.doc]

**Additional file**

**TABLE S1. Percentiles of WC, WHtR and BMI in men (n=1924) and women (n=1825)**

|  |  | **20th** | **40th** | **50th** | **60th** | **80th** |
| --- | --- | --- | --- | --- | --- | --- |
| **WC** | **men** | 85.80 | 92.80 | 95.50 | 98.20 | 105.20 |
|  | **women** | 71.15 | 78.10 | 81.50 | 85.50 | 94.50 |
|  | | | | | | |
| **WHtR** | **men** | 0.49 | 0.53 | 0.55 | 0.57 | 0.61 |
|  | **women** | 0.43 | 0.48 | 0.50 | 0.53 | 0.59 |
|  | | | | | | |
| **BMI** | **men** | 24.27 | 26.50 | 27.41 | 28.33 | 30.75 |
|  | **women** | 22.31 | 24.61 | 26.16 | 27.54 | 31.33 |

BMI = body-mass index; WC = waist circumference; WHtR = waist-to-height ratio.

**TABLE S2.** Logistic regression models for the associations between sex-specific quintiles of WC, WHtR or BMI and microalbuminuria or CKD in the subjects time of examination before noon (n = 2712).

|  | **Microalbuminuria** | | **CKD** | |
| --- | --- | --- | --- | --- |
|  | **OR (95 % CI)** | **p-value** | **OR (95 % CI)** | **p-value** |
| ***Waist circumference (WC) [cm]*** | | | | |
| I | **1.55 (1.06; 2.28)** | **0.03** | 1.12 (0.58; 2.18) | 0.74 |
| II | 1.06 (0.74; 1.52) | 0.73 | 1.08 (0.65; 1.81) | 0.77 |
| III | *reference* |  | *reference* |  |
| IV | 1.23 (0.90; 1.69) | 0.19 | 1.23 (0.80; 1.87) | 0.35 |
| V | **1.62 (1.19; 2.19)** | **<0.01** | **1.60 (1.06; 2.43)** | **<0.01** |
| ***Waist-to-height ratio (WHtR)*** | | | | |
| I | 1.21 (0.80; 1.83) | 0.38 | 1.37 (0.62; 3.05) | 0.44 |
| II | 0.89 (0.62; 1.28) | 0.54 | 1.41 (0.82; 2.42) | 0.22 |
| III | *reference* |  | *reference* |  |
| IV | 1.13 (0.83; 1.55) | 0.43 | 1.31 (0.84; 2.05) | 0.23 |
| V | **1.39 (1.03; 1.88)** | **0.03** | **1.96 (1.28 2.99)** | **<0.01** |
| ***Body mass index (BMI) [kg/m^2^]*** | | | | |
| I | 1.28 (0.90; 1.84) | 0.17 | 0.85 (0.47; 1.54) | 0.60 |
| II | 0.88 (0.62; 1.25) | 0.49 | 0.76 (0.45; 1.27) | 0.29 |
| III | *reference* |  | *reference* |  |
| IV | 1.00 (0.73; 1.37) | 0.99 | 0.94 (0.61; 1.44) | 0.76 |
| V | 1.23 (0.90; 1.67) | 0.19 | 1.48 (0.97; 2.24) | 0.07 |

CI = confidence interval; CKD = chronic kidney disease; OR = odds ratio

**TABLE S3.** Logistic regression models for the associations between sex-specific quintiles of WC, WHtR or BMI and microalbuminuria in the whole study population (n = 3749).

|  | **original model** | |  | **+ HDL cholesterol** | |  | **+ total cholesterol** | |
| --- | --- | --- | --- | --- | --- | --- | --- | --- |
|  | **OR (95 % CI)** | **p-value** |  | **OR (95 % CI)** | **p-value** |  | **OR (95 % CI)** | **p-value** |
|  |  |  |  |  |  |  |  |  |
| ***Waist circumference (WC) [cm]*** | | | | | | | | |
| I | **1.48 (1.06; 2.06)** | **0.02** |  | **1.53 (1.09; 2.14)** | **0.01** |  | **1.50 (1.07; 2.09)** | **0.02** |
| II | 1.14 (0.84; 1.55) | 0.40 |  | 1.14 (0.84; 1.56) | 0.40 |  | 1.11 (0.82; 1.51) | 0.50 |
| III | *reference* |  |  | *reference* |  |  | *reference* |  |
| IV | 1.21 (0.91; 1.60) | 0.18 |  | 1.17 (0.89; 1.56) | 0.26 |  | 1.18 (0.89; 1.57) | 0.24 |
| V | **1.52 (1.16; 2.00)** | **<0.01** |  | **1.48 (1.13; 1.94)** | **<0.01** |  | **1.53 (1.17; 2.01)** | **<0.01** |
|  |  |  |  |  |  |  |  |  |
| ***Waist-to-height ratio (WHtR)*** | | | | | | | | |
| I | 1.20 (0.85; 1.71) | 0.30 |  | 1.25 (0.88; 1.78) | 0.22 |  | 1.23 (0.87; 1.75) | 0.24 |
| II | 0.91 (0.66; 1.24) | 0.54 |  | 0.92 (0.67; 1.26) | 0.60 |  | 0.90 (0.66; 1.23) | 0.50 |
| III | *reference* |  |  | *reference* |  |  | *reference* |  |
| IV | 1.06 (0.80; 1.39) | 0.69 |  | 1.04 (0.79; 1.37) | 0.80 |  | 1.05 (0.80; 1.38) | 0.74 |
| V | **1.33 (1.03; 1.74)** | **0.03** |  | **1.31 (1.00; 1.71)** | **0.05** |  | **1.34 (1.03; 1.75)** | **0.03** |
|  |  |  |  |  |  |  |  |  |
| ***Body mass index (BMI) [kg/m^2^]*** | | | | | | | | |
| I | 1.28 (0.94; 1.75) | 0.11 |  | 1.34 (0.98; 1.84) | 0.07 |  | 1.29 (0.94; 1.76) | 0.12 |
| II | 0.91 (0.68; 1.23) | 0.56 |  | 0.93 (0.69; 1.27) | 0.66 |  | 0.92 (0.68; 1.24) | 0.59 |
| III | *reference* |  |  | *reference* |  |  | *reference* |  |
| IV | 1.01 (0.76; 1.33) | 0.96 |  | 1.01 (0.77; 1.34) | 0.93 |  | 1.02 (0.78; 1.35) | 0.87 |
| V | 1.16 (0.89; 1.53) | 0.27 |  | 1.14 (0.86; 1.49) | 0.36 |  | 1.18 (0.90; 1.55) | 0.23 |

CI = confidence interval; HDL = high-density lipoprotein; OR = odds ratio.

**TABLE S4.** Logistic regression models for the associations between sex-specific quintiles of WC, WHtR or BMI and CKD in the whole study population (n = 3749).

|  | **original model** | |  | **+ HDL cholesterol** | |  | **+ total cholesterol** | |
| --- | --- | --- | --- | --- | --- | --- | --- | --- |
|  | **OR (95 % CI)** | **p-value** |  | **OR (95 % CI)** | **p-value** |  | **OR (95 % CI)** | **p-value** |
|  |  |  |  |  |  |  |  |  |
| ***Waist circumference (WC) [cm]*** | | | | | | | | |
| I | 1.26 (0.75; 2.11) | 0.38 |  | 1.48 (0.88; 2.51) | 0.14 |  | 1.27 (0.76; 2.14) | 0.36 |
| II | 1.12 (0.73; 1.71) | 0.60 |  | 1.23 (0.80; 1.89) | 0.34 |  | 1.11 (0.73; 1.70) | 0.63 |
| III | *reference* |  |  | *reference* |  |  | *reference* |  |
| IV | 1.19 (0.83; 1.71) | 0.35 |  | 1.18 (0.82; 1.70) | 0.37 |  | 1.20 (0.83; 1.72) | 0.33 |
| V | **1.63 (1.15; 2.32)** | **<0.01** |  | **1.55 (1.09; 2.21)** | **0.02** |  | **1.65 (1.16; 2.34)** | **<0.01** |
|  |  |  |  |  |  |  |  |  |
| ***Waist-to-height ratio (WHtR)*** | | | | | | | | |
| I | 1.56 (0.87; 2.78) | 0.14 |  | **1.80 (1.00; 3.25)** | **0.05** |  | 1.57 (0.88; 2.81) | 0.13 |
| II | 1.39 (0.89; 2.17) | 0.14 |  | 1.54 (0.98; 2.42) | 0.06 |  | 1.37 (0.87; 2.14) | 0.17 |
| III | *reference* |  |  | *reference* |  |  | *reference* |  |
| IV | 1.18 (0.81; 1.72) | 0.39 |  | 1.16 (0.80; 1.70) | 0.44 |  | 1.17 (0.81; 1.71) | 0.40 |
| V | **1.83 (1.28; 2.60)** | **<0.01** |  | **1.72 (1.20; 2.45)** | **<0.01** |  | **1.84 (1.29; 2.61)** | **<0.01** |
|  |  |  |  |  |  |  |  |  |
| ***Body mass index (BMI) [kg/m^2^]*** | | | | | | | | |
| I | 0.92 (0.57; 1.48) | 0.72 |  | 1.03 (0.63; 1.67) | 0.90 |  | 0.89 (0.55; 1.43) | 0.62 |
| II | 0.86 (0.56; 1.31) | 0.48 |  | 0.91 (0.59; 1.38) | 0.65 |  | 0.86 (0.56; 1.30) | 0.47 |
| III | *reference* |  |  | *reference* |  |  | *reference* |  |
| IV | 1.00 (0.70; 1.44) | 0.99 |  | 0.98 (0.68; 1.41) | 0.91 |  | 1.00 (0.69; 1.44) | 1.00 |
| V | **1.47 (1.04; 2.10)** | **0.03** |  | 1.40 (0.98; 1.99) | 0.07 |  | **1.48 (1.04; 2.11)** | **0.03** |

CI = confidence interval; CKD = chronic kidney disease; HDL = high-density lipoprotein; OR = odds ratio.
